# Learning of Multi-Modal Stimuli in Hawkmoths

**DOI:** 10.1371/journal.pone.0071137

**Published:** 2013-07-29

**Authors:** Anna Balkenius, Marie Dacke

**Affiliations:** 1 Department of Chemical Ecology, Swedish University of Agricultural Science, Alnarp, Sweden; 2 Department of Biology, Lund University, Lund, Sweden; Monash University, Australia

## Abstract

The hawkmoth, *Manduca sexta*, uses both colour and odour to find flowers when foraging for nectar. In the present study we investigated how vision and olfaction interact during learning. *Manduca sexta* were equally attracted to a scented blue coloured feeding target (multimodal stimulus) as to one that does not carry any scent (unimodal stimulus; visual) or to an invisible scented target (unimodal stimulus; odour). This naive attraction to multimodal as well as to unimodal stimuli could be manipulated through training. Moths trained to feed from a blue, scented multimodal feeding target will, when tested in a set-up containing all three feeding targets, select the multimodal target as well as the scented, unimodal target, but ignore the visual target. Interestingly, moths trained to feed from a blue, unimodal visual feeding target will select the visual target as well as the scented, multimodal target, but ignore the unimodal odour target. Our results indicate that a multimodal target is perceived as two separate modalities, colour and odour, rather than as a unique fused target. These findings differ from earlier studies of desert ants that perceive combined visual and odour signals as a unique fused stimulus following learning trials.

## Introduction

The world around us is rich in multisensory stimuli, such as a scented flower or a hairy fruit. These cross-modal stimulus combinations are often conveyed by different forms of energy and transduced by independent sensory systems [1]. Since different modes of sensory input (for example from a visual or scented stimulus) are not influenced by the same sources of noise, a combination of signals conveyed by different senses reduces the risk of incorrect information processing [1], [2]. Multisensory redundancy allows animals to reliably detect and correctly identify prey species or flowers rich in nectar. A recent study on *Drosophila* demonstrates how cross-modal stimulus combinations influence flight behaviour already at some distance from the target [3]. In these flies, an attractive odour perceived in flight will increase the influence of yaw-optic flow in such a way that it will result in a straighter flight path compared to the flight path without olfactory information.

The hawkmoth *Manduca sexta* uses both colour and odour to locate a nectar flower [4], [5]. Day active hawkmoths rely primarily on visual input to forage [6], while hawkmoths that are active at low light levels (such as *M. sexta*) rely mainly on odour [6], [7], even though they perceive colour under low light levels [8]. *Manduca sexta* has an innate preference for blue flowers [9] but is most often observed foraging from white or yellow night-blooming flowers in the wild [10], [11]. These moths can also be trained to select an initially disregarded flower colour, such as green [12]. Hawkmoths migrate over long distances, and the colour and odour of suitable nectar flowers vary across the different habitats they encounter. It is thus advantageous that the detection of suitable flowers to feed from can be modified through learning [12], [13].

Previous studies on *M. sexta* demonstrate complex interactions of colour and odour on behaviour and brain activity [14], [12], [6], [5]. When approaching a scented and clearly visible feeding target (multimodal stimulus) the moths turn towards the target and decrease their flight speed earlier than if the clearly visible feeding target carries no scent (unimodal stimulus) [12]. If approaching a scented but effectively invisible feeding target, the moths fail to adequately decrease their flight speed and overshoot or crash into the target. Scent alone can thus guide the moth to the location of the flower, but visual information is necessary for the moth to accurately slow down in front of the feeding target [4]. Neural integration of colour and odour signals occurs in the mushroom body of the hawkmoth [14], and activity in this part of the brain has been demonstrated to vary with the colours and odours that are presented to the moth. For example, during training to a flower odour (phenylacetaldehyde) activity in the mushroom body changes in response to a rewarded stimulus configuration. Interestingly, the response latency to a multimodal stimulus is always shorter than the reaction to a unimodal stimulus [15].

Recent studies on ants show that training with a multimodal nest entrance marker (colour and odour) initially evokes a response to the two unimodal subsets of this stimulus. After 15 training runs theses responses secede and the ants responded only to the multimodal stimulus [16]. In this study we investigate whether the hawkmoth *M. sexta* follows the same sensory learning process as that of the ant. Specifically, we ask whether a moth trained to a multimodal stimulus (a scented, blue feeding target) will continue to feed from unimodal feeding targets (blue or scented targets) and how these preferences change over time.

## Results

### Preference of naive and unrewarded hawkmoths

Naive moths (group 1) were released in the circular flight arena (diameter 1.5 m) with three feeding targets to chose from: a visual feeding target composed of a flower-like blue feeder (V); a transparent feeding target scented with bergamot oil (O); and a multimodal feeding target composed of a visual target scented with bergamot oil (M). The first feeder choice of each moth was recorded. A choice was deemed to have been made when the moth hovered or slowed down in front of a feeding target. No reward was present during the tests.

**Figure 1:**
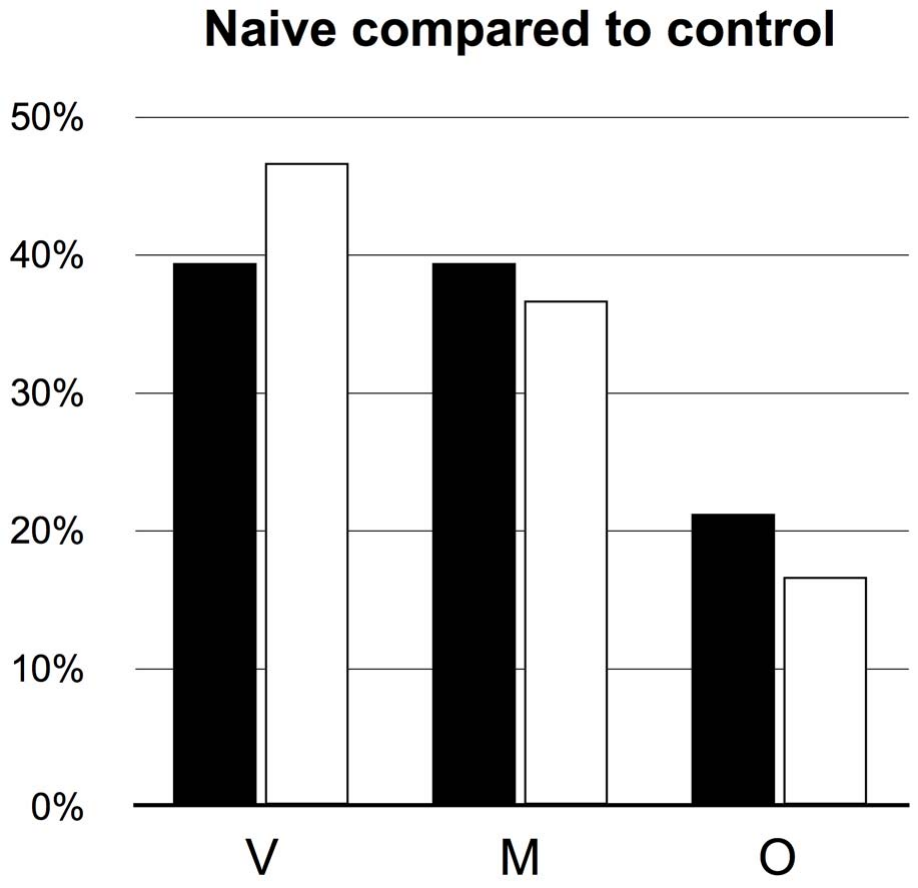
Feeding preference of naive and control moths. Proportion of choices for the three different feeding targets; multimodal (M), visual (V) and scented (O) by naive moths and the control group of moths (handled in the same way as the rewarded animals, but without any reward).

As a control for possible effects from handling the moths, an unrewarded group of moths were handled in the same way as the rewarded animals in the experiments below, but without any reward, i.e. their proboscis was unrolled into an empty feeding target with pale white as the background. The feeding preference of this control group of moths was not significantly different from that of the naive moths (Fisher's exact test, P = 0.85, n = 33; Fig. 1).

### Preference of moths trained to a multimodal feeding target (M+)

**Figure 2:**
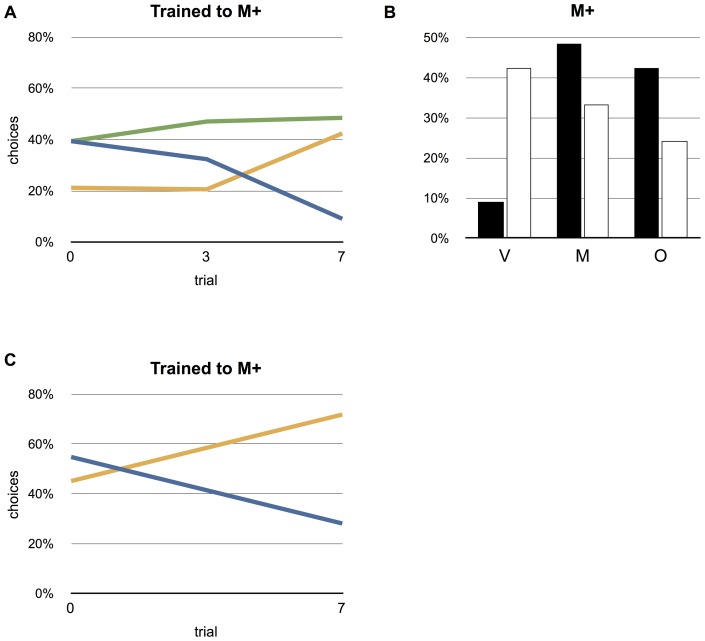
Effect of training to the multimodal target (M+). (A) Choices before training and after three and seven rewards with the animals that were trained to the multimodal stimulus (M+) and tested in an arena with three feeding targets (multimodal (M), visual (V) and scented (O)). (B) Choices after training compared to the control group. (C) The animals where trained with the multimodal stimulus (M+) and tested with only the two unimodal feeding targets (V) and (O) after receiving seven rewards.

Moths rewarded three times from the multimodal feeding target (M+) (group 2) did not select any of the feeding targets (M, V and O) in the flight arena over the others, and the feeding preference of these moths did consequently not differ from that of the unrewarded control group (Fisher's exact test, P = 0.86, n = 34; Fig. 2A). However, after the moths had been rewarded seven times from the multimodal feeding target (M+) (group 3) their feeding preference differed significantly from that of the unrewarded control group (P<0.05, n = 33; Fig. 2A, B). Following the seventh reward the visual feeding target (V) of group 3 was selected less than that of the control group or moths rewarded only three times (group 2).

This pattern of learning was similar to that observed when the moths were tested in an arena with only two feeding targets (V and O) present. The choice frequency of hawkmoths trained seven times to M+ and tested in the arena with only two stimuli present, V and O (group 6), differed significantly in their feeding preference from the control group (P<0.05, n = 32; Fig. 2C). While the control group of moths selected the two feeding targets equally often, the moths trained to the multimodal feeding target selected the invisible, scented target (O) almost four times as often as they selected the visible target (V).

### Preference of moths trained to blue feeding target (V+)

**Figure 3:**
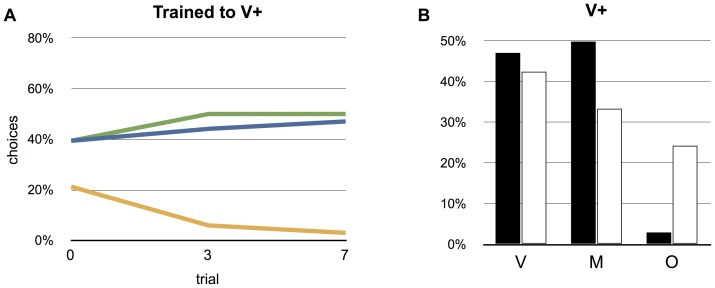
Effect of training to a unimodal, visual target (V+). (A) Choices before training and after three and seven rewards for the animals trained with the visual stimulus (V+) and tested in the arena with three feeding targets (multimodal (M), visual (V) and scented (O)). (B) Choices after training compared to the control group.

The feeding preferences of hawkmoths trained three times to the blue, unscented feeding target (V+) (group 4), and then tested in the arena with the three stimuli M, V and O present did not differ significantly from that of the unrewarded control group (group 7) (Fisher's exact test, P = 0.2, n = 34) (Fig. 3A). In contrast, the hawkmoths that were trained seven times to V+ (group 5) before being released into the arena had a feeding preference that was significantly different from that of the unrewarded control group of moths (P<0.05, n = 34, n = 33) (Fig. 3B). The moths then ignored the invisible, scented target (O), but continued to approach both the unimodal visible target (V) and the multimodal target (M).

### Proboscis extension, choice latency and effect of handling

**Figure 4:**
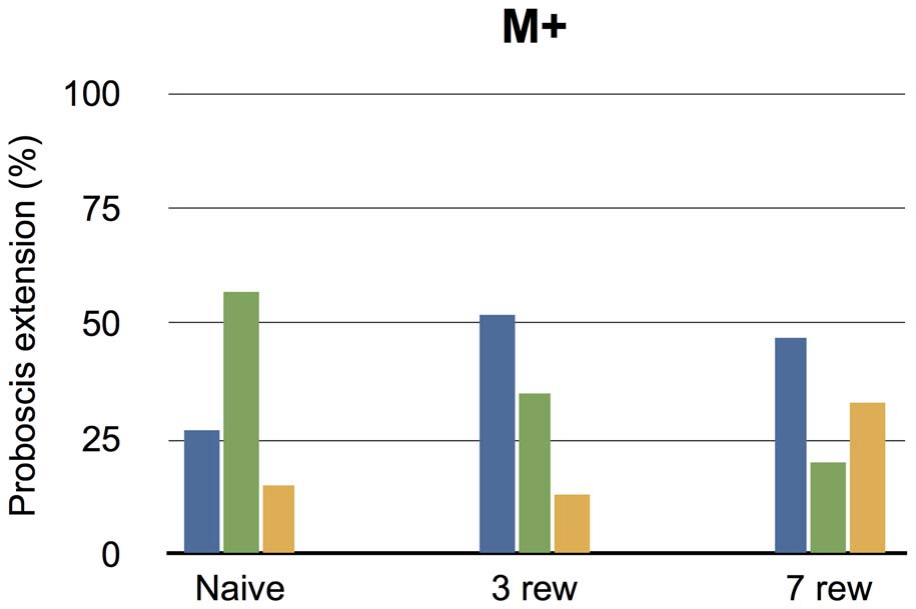
Proboscis extensions. The percentage of proboscis extensions in front of a the multimodal (M), visual (V) or scented (O) feeding target after training to the multimodal target (M+).

The proportion of proboscis extensions in front of the different feeding targets was recorded for naive moths (group 1) and for the groups of moths that were fed three or seven times from the multimodal feeding target (group 2, 3). In general, the moths extended their proboscis more when there was a visual feeding target in front of them (M or V) than when the target was scented but effectively invisible (O) (Fig. 4). We found no significant difference between the proportions of proboscis extension in front of the different feeding targets for the naive and the three times rewarded groups of moths (Fisher's exact test, P = 0.08), but there was a significant difference in the proportions of proboscis extensions between the unrewarded handling control group of animals and the group that had been rewarded seven times (Fisher's exact test, P<0.05). Even though this group of moths had been frequently rewarded from the multimodal feeding target, the training also had a stimulating effect on the proportion of proboscis extensions in front of the invisible, scented target (O).

The choice latency of each moth was defined as from when the moth started to fly in the arena until it slowed down or hovered in front of one of the feeding targets. The choice latency for the different feeding targets varied for naive (V = 20±2 s, O = 13±3 s, M = 15±3 s), three times rewarded (V = 16±3 s, O = 11±3 s, M = 19±3 s) and seven times rewarded moths (V = 19±4 s, O = 18±3 s, M = 15±4 s), but the average response time of rewarded moths did not differ significantly from that of the naive moths (Fisher's exact test; three times rewarded P = 0.51, seven times rewarded P = 0.62).

## Discussion

Desert ants can be trained to rely on the position of a clearly visible, black cardboard landmark scented with indole to identify the position of its nest [16]. After training, this multimodal target is treated as one fused stimulus, and the individual stimuli (vision and odour) are ignored when presented on their own. Several studies have shown that *M. sexta* can use colour or odour individually, and their combination, to find flowers [4], [5], [17], [18], but the question about how they separate or fuse these different modalities have never been tested. Here we show that *M. sexta* do not treat a multimodal stimulus as one fused cue even after several rewarded trials.

After seven rewards to the multimodal feeding target (a scented visual stimulus, M) the moths preferentially fly towards this target as well as toward an invisible unimodal scented target (O) (Fig. 2). The visible, unscented feeding target (V), presented together with these two targets, is largely ignored. If the moths like the ants had learned the fused combination, both unimodal feeding targets (V and O) would have been ignored. The preference for the scented targets (M and O) after training is likely due to *M. sexta,* initially using odour to locate the general area of the feeding station, followed by visual homing [4]. In other words, the moth is guided towards a flower by its scent even if it can not yet see it. Moths trained to the multimodal targets selected the scented target over the visual target in an arena with only a unimodal visual target (V) and unimodal scented target (O) to choose from. Again, this indicates that the trained moth does not ignore the individual stimulus modalities (vision and scent) even after repeated training to the multimodal stimulus. In this set-up, many of the moths started to fly towards the scented target and slowed down, but then gave up and landed on the arena floor. This could possibly be an effect of not finding the visual information to indicate the exact position of the feeding target.

The same outcome as above would have been reached if the moths simply treated the multimodal feeding target as a unimodal scented target, and ignored the visual information. However, the behaviour of moths trained to the unimodal visual target (V) shows that this is not the case. When tested in an arena with all three feeding targets present (V, O and M) the moths trained to the blue visual target (V+) selected this feeding target (V) as well as the multimodal target (M), but ignored the unimodal invisible scented target (O). In this case, the moths had likely learned that visual cues, but not odour, were important and clearly identified this as a separate modality of the multimodal stimulus (Fig. 2B).

To test for a possible effect of handling on the moths during training (the moth was held in front of the rewarded feeding target and the proboscis manually unrolled to stimulate feeding from the rewarded target), we manipulated a group of moths in the same way as during training but used no reward. When released in the arena, these unrewarded control moths did not differ in their target preference from naive moths. Thus, the changed target preference of the moths trained to the multimodal (M+) or the visual (V+) feeding target was a result of the repeated reward and not the handling of the moths (Fig. 2 and 3). Interestingly, trained moths did not become faster in locating the feeding target. An explanation for this could be that the flight in the test arena is the first flying experience for all moths irrespective of training experience, and that it takes some time before the moths start to explore the arena for nectar.

*M. sexta* have been observed to shift their colour preference after only one learning trial [12], but when trained to the multi-sensory feeding target used in this study we did not record any significant effects until after four to seven trials. These results agree with a recent study measuring the activity in the brain during training to a multimodal stimuli [15], where it was reported that training moths to a visible and scented multimodal stimuli training changed the response of the mushroom body.

Even though we observed a clear effect on target preference in moths repeatedly trained to a multimodal feeding target, the moths did not treat the colour and odour of this target as a single fused stimulus. In contrast, ants treat a multimodal target as a fused target after repeated training. This difference in stimulus fusion could be an effect of the number of learning trials, as the ants were exposed to fifteen learning trials while the moths were only exposed to seven learning trials (this was the upper limit to how long we could starve the newly hatched moths before we detected a decrease in the willingness to fly). Since the moths displayed a clear learning effect after seven learning trials, we consider the difference in the number of learning trials an unlikely explanation for the difference in stimuli fusion between the moths and the ants. The differences in stimuli fusion between the two species is more likely a result of the vastly different behavioural tasks the animal were tested for, namely homing and feeding. For a highly precise homing task the nest is always at the same place, carrying the same scent, while for feeding the occurrences and type of food will vary. It may then be more flexible and highly advantageous to remain sensitive to the individual stimuli modalities, rather than to treat the feeding target as one fused stimulus. Since *M. sexta* do not home to a precise locality we could not test for such a task dependent difference in modality fusion.

## Conclusions

In conclusion, we show that *M. sexta* do not fuse odour and colour into a single unitary stimulus even after several training sessions. Instead, they react as if they had learned the significance of each modality independently. Such a non-fused sensory system would support a flexible feeding system over a highly precise one, and would be beneficial to a migrating moth that frequently changes its feeding area.

## Materials and Methods

### Animals and environment

Larvae of the hawkmoth *Manduca sexta* (Lepidoptera: Sphingidae) were reared on an artificial diet [19] with 200 mg beta-carotene L^−1^ added [22]. The animals were kept under a 16∶8 h light:dark cycle at 23–25°C, 40–50% relative humidity. Both male and female moths were used in the experiment, 3–5 days after eclosure. The experiments were conducted in a circular arena with a diameter of 1.5 m, surrounded by a 0.5 m wall and covered with a transparent net. Individual moths were released into the arena from the centre of the arena floor. The light intensity in the visible range was 0.95 cd m^−2^, a level that the moth typically encounters in the wild [20] and also one which allowed the camera system to operate.

The visual feeding target (V) was composed of a flower-like blue feeder [21], and the scented feeding target (O) consisted of a transparent, scented thin glass tube (1.5 mm diameter). The blue target reflects preferentially at a wavelength of 450 nm. Bergamot oil (5 μl) (Aroma, essential oil *Citrus aurantium bergamia*, Italy) was used to scent the targets. This oil contains linalool and monoterpenoid odours that are released by many night-blooming flowers [23] and has previously been used to stimulate feeding in *M. sexta* [4], [5], [11]. The odour source was replaced every hour to avoid depletion. To control for the visibility of the scented feeding target, the arena was fitted with three unscented glass tubes placed well apart from each other. Out of the eight moths that were flown in the arena one at the time, none of them made any apparent attempts to avoid the transparent tubes and three moths also collided with the transparent tubes in mid air. The multimodal feeding target (M) was composed of a visual target scented with bergamot oil.

The shape and the position of the odour plume from the multimodal and the scented feeding target were simulated by applying dry ice at the same location as the odour source in the arena. This established that there were no air currents in the arena and that the concentration of the applied odour was only influenced by passive diffusion and the turbulence from the flying moth. Flights of moths in the arena were recorded with a low light capable video camera (Sony HDR-CX11E, Japan) and wide conversion lens 0.42X KCW-04212.5.

### Experimental protocol

The naive animals were divided into eight groups: *1*: The first group was used for preference tests and released into the cage without being pre-exposed to any colour or odour. *2,3*: Two groups were trained to feed from a rewarded multimodal feeding target (M+), three or seven times respectively and subsequently released into the arena with the three different stimuli (V, O and M) presented in a triangle 50 cm apart. To not injure the moths during training, they were manually held in front of the feeding target while preventing movements of the wings and legs. The proboscis was carefully unrolled with the tip of a pipette to stimulate feeding of a 20% sugar solution from the rewarded feeding target [12]. Between trials, they were placed in a small canister that prevented flight. *4,5*: Two groups were trained to feed from a rewarded unimodal visual feeding target (V+), three or seven times respectively and subsequently released into the arena with the different modalities present as above. *6*: One group was trained to feed from a rewarded multimodal feeding target (M+) seven times and released into the arena with only V and O present, placed 50 cm apart. *Control*: As a control for possible effects from handling the moths, an unrewarded group of moths were handled in the same way as the rewarded animals in group 2–7, but without any reward, i.e. their proboscis was unrolled into an empty cup but with pale white as the background.

In all experiments, the moths warmed up for at least 10 minutes and were then allowed to fly until they approached a target or had flown for a maximum of 3 minutes. Each moth was tested individually and its first choice of stimulus was recorded. It was considered a choice when the moth hovered in front of the feeding target and extended its proboscis in an attempt to feed, or slowed down close to the scented feeding target while oriented towards it. No reward was present during the tests. The animals were then removed from the arena and were not used again. Animals that did not fly, or landed before they had approached a target were discarded from any further participation in the experiments. After take-off, the moths typically flew upwards and stabilised their flights approximately 10 cm below the ceiling of the arena. Every training and test session included all seven groups to avoid an effect of daily changes in motivation.
